# Nitazoxanide alleviates CFA-induced rheumatoid arthritis in Wistar rats by modulating the STAT-3 and NF-κB pathways

**DOI:** 10.1515/rir-2025-0004

**Published:** 2025-04-02

**Authors:** Pradyuman Prajapati, Gaurav Doshi

**Affiliations:** Department of Pharmacology, SVKM’s Dr Bhanuben Nanavati College of Pharmacy, Vile Parle (W), Mumbai, India

**Keywords:** rheumatoid arthritis, nitazoxanide, interlukin-6, signal transducer and activator of transcription 3, nuclear factor kappa B

## Abstract

**Background and Objective:**

Rheumatoid arthritis (RA) is a chronic autoimmune condition characterized by joint pain and inflammation. RA involves elevated expression of nuclear factor kappa B (NF-κB), interleukin-6 (IL-6), and tumor necrosis factor-alpha (TNF-α), which drive synovial inflammation and joint destruction primarily through the STAT-3 signalling pathway. Nitazoxanide (NTZ) has been shown in previous studies to inhibit the signalling of STAT-3.

**Methods:**

This study evaluated the anti-arthritic effects of NTZ in a rat model of complete Freund’s adjuvant (CFA) induced arthritis. NTZ was administered orally at doses of 400, 200, and 100 mg/kg over 28 days. Various parameters, including changes in paw swelling, body weight, arthritic index, haematological measurements, levels of inflammatory cytokines, and histopathological analysis, were monitored.

**Results:**

NTZ treatment significantly improved body weight and reduced paw swelling, edema, and the arthritic index in CFA-induced arthritic rats. The treatment also decreased white blood cell counts while increasing red blood cell and haemoglobin levels. NTZ effectively modulated inflammatory cytokine levels and showed improvement in the histopathology of the ankle joints.

**Conclusion:**

NTZ exhibited significant anti-arthritic activity through the inhibition of the STAT-3 and NF-κB pathways, emphasizing its potential as a therapeutic option for rheumatoid arthritis.

## Introduction

Rheumatoid arthritis (RA) is a rapidly progressing chronic autoimmune disorder. It causes swelling, pain, and inflammation in and around the joints, and as the disease advances, it begins to damage the tendons and ligaments throughout the body.^[1,2]^ As of 2019, an estimated 18 million people across the globe were affected by RA.^[3]^ Clinical reports indicate that about 0.5%–1% of the global population is affected by RA, with approximately 25 to 50 new cases emerging per 100, 000 people annually.^[4]^ Previous reports indicate that patients suffering from RA experience negative impacts on their daily routines, often finding it difficult or impossible to complete regular tasks. These challenges can significantly affect their quality of life.^[5,6]^ RA leads to joint destruction by increasing the expression of transcription factors and cytokines.^[7,8]^ Inflammatory cytokines such as interleukin-6 (IL-6), nuclear factor kappa B (NF-κB) and tumour necrosis factor-alpha (TNF-α), play a key role in the development and progression of RA.^[9,10]^ TNF-α enhances the inflammatory response by promoting the migration of synovial fibroblasts and leukocytes, as well as increasing the expression of cellular adhesion molecules within the inflamed joint.^[11]^ IL-6 enhances the inflammatory response by facilitating the expansion of blood vessels. RA is a chronic autoimmune disorder in which IL-6 plays a critical role in immune activation and inflammation. Consequently, inhibiting IL-6 presents a promising strategy for managing RA.^[12]^ As a result, the imbalance between inflammatory cytokines and anti-inflammatory states has led to joint damage and the induction of inflammation in the synovial membrane.^[5,6]^ The therapeutic management of RA has largely concentrated on alleviating symptoms due to various etiological factors, including inflammation, preventing tissue damage, and maintaining function. Available treatment options include analgesics, Non-steroidal Anti-Inflammatory Drugs (NSAIDs), glucocorticoids, Disease-Modifying Antirheumatics Drugs (DMARDs), and anti-cytokine therapies, which can be administered individually or in combination. However, these options are often inadequate for effectively treating RA. Therefore, there is an urgent need to discover an appropriate drug that can successfully manage RA symptoms.

The signal transducer and activator of transcription 3 (STAT-3) is a transcription factor found in the cytoplasm that gets activated in response to inflammatory cytokines or growth factors. Once activated, it translocates to the nucleus and regulates DNA transcription.^[13]^ STAT-3 has attracted significant research focus due to its crucial involvement in various biological processes, such as cell proliferation, differentiation, anti-apoptotic mechanisms, inflammatory responses, and angiogenesis.^[14, 15, 16]^ Cytokines have been associated with the progression of RA, as demonstrated by a significant rise in pro-inflammatory cytokines such as TNF-α, and IL-6. These cytokines influence RA primarily through the STAT-3 signalling pathway. For example, IL-6 can activate the STAT-3 pathway, while TNF-α can also activate this pathway by inducing the phosphorylation of STAT-3.^[17,18]^

Nitazoxanide (NTZ) is an antiparasitic drug from the thia-zolide class. It has been approved by the U. S. Food and Drug Administration (FDA) for the treatment of *Cryptosporidium parvum* and *Giardia intestinalis* infections in children and adults.^[19]^ Recent research has revealed that NTZ showcases a diverse spectrum of pharmacological effects, demonstrating efficacy against both infectious diseases and oncological disorders.^[20, 21, 22]^ A recent study has identified NTZ as a potent inhibitor of STAT-3, effectively blocking the activation of the STAT-3 pathway in cancer cells.^[23,24]^ STAT-3 plays a crucial role in embryonic development, as demonstrated by STAT-3 global knockout mice experiencing early embryonic lethality. However, adult mice do not exhibit lethality when STAT-3 is globally inhibited, indicating that STAT-3 could be a viable therapeutic target in adults. Since overproduction of IL-6 is commonly observed in patients and models of RA, blocking IL-6 activity may offer therapeutic benefits in treating RA. In this context, Tomoaki Mori *et al*. investigated the potential of STAT-3 inhibitors and demonstrated their ability to disrupt the inflammatory cytokine IL-6 loop, which drives STAT-3 activation.^[25]^ Earlier research has shown that NTZ acts as an inhibitor of the STAT-3 signalling pathway, with the ability to restrict cancer cell growth and decrease bone loss in a model of ovariectomized mice.^[23,26]^ The therapeutic approach aimed at targeting intercellular STAT-3 and NF-κB signalling pathways has transformed the current landscape of RA treatment.^[27]^ The median lethal dose (LD50) values for male and female rats were found to be greater than 10 g/kg. A 14-week oral toxicity study in rats showed no systemic effects or blood-related abnormalities at daily doses of up to 450 mg drug per kg of body weight, suggesting that repeated treatment would be safe.^[28]^ NTZ was administered orally at doses of 100, 200, and 400 mg/kg daily over 28 days. Key parameters were evaluated, including body weight, paw swelling, arthritic index, haematological changes, inflammatory cytokine levels, joint histopathology, and radiography images. The complete Freund’s adjuvant (CFA)-induced arthritis model is widely used to evaluate the effectiveness of treatments for both acute and chronic inflammation, with IL-6 expression playing a pivotal role in driving the inflammatory process. Chosen for its resemblance to human arthritis, the model mimics key features such as bone erosion, cartilage destruction, vascular formation, and synovial hyperplasia. CFA comprises heat-killed *Mycobacterium tuberculosis* and liquid paraffin, triggering a cellular immune response and antibody production, leading to inflammation within 3–5 days. Rat paw swelling offers a sensitive measure for assessing inflammation and treatment efficacy, making CFA a preferred model over collagen-induced arthritis (CIA) for its closer simulation of human arthritic conditions.^[29]^ The signalling pathways targeted by NTZ reveal their mechanisms of action in reducing inflammation. This study reported that NTZ-induced inhibition of IL-6 is connected to the simultaneous suppression of STAT-3 and NF-κB activity. Notably, NTZ treatment resulted in the deactivation of both STAT-3 and NF-κB and reduced IL-6 expression in CFA-induced paw tissues, suggesting its ability to modulate these transcription factors in inflammation, regardless of the triggering stimulus.

## Materials and Methods

### Chemical and Drugs

Nitazoxanide was provided as a gift sample by Nirali Life Sciences Pvt. Ltd., Gujarat, India. Prednisolone was also received as a gift sample from Avik Pharmaceutical Ltd., Gujarat, India, while CFA was sourced from Sigma Aldrich, St. Louis, Missouri, USA. All additional reagents utilized in the experiment were of analytical grade. Enzyme-linked immunosorbent assay (ELISA) kits for assessing serum cytokine levels were supplied by Allianz Bioinnovation Pvt. Ltd.

### Animals

The National Institute of Biosciences in Pune, India provided healthy Wistar rats weighing between 150 and 180 grams. The rats were accommodated in plastic Perspex cages under controlled conditions, with the temperature set at 25°C, relative humidity maintained between 45%–55%, and a 12-hour light-dark cycle. They were provided with unrestricted access to chow pellets and purified water. Before commencing the experiments, the rats were allowed a one-week acclimatization period in their new environment. The experimental protocol was reviewed and approved by the Institutional Animal Ethics Committee (CCSEA/IAEC/P-67/2023), which is registered under the “Committee for Control and Supervision of Experiments on Laboratory Animals (CCSEA)” of the Ministry of Environment and Forests, Government of India.

### CFA‑induced Arthritic Model

Subcutaneous injection of Complete Freund’s Adjuvant into the subplantar region of the left hind paw of rats caused inflamed swelling (primary lesion) within 30 min, with peak inflammation occurring within 3–4 days. Secondary lesions appeared at non-injected sites, including the non-injected hind paw, forepaws, ears, nose, and tail, between 12–14 days post-injection. The CFA-induced joint inflammation generally persisted for 25–28 days.

### Experimental Design

On day 0, a total of 0.1 mL of CFA was administered into the subplantar area of the left hind paw for all experimental groups, except for the normal control group, which was administered paraffin oil in an amount of 0.1 mL. Carboxymethyl Cellulose (CMC) served as the medium for the preparation of Prednisolone acetate.^[30]^ All groups received treatments through oral administration for 28 days. Animals were divided into 6 groups with 6 rats in each group (*n* = 6): Group 1, Normal Control (Normal Saline); Group 2, Disease Control (CFA with no treatment); Group 3, Standard Control (Prednisolone 10 mg/kg); Group 4, Nitazoxanide (100 mg/kg); Group 5, Nitazoxanide (200 mg/kg); Group 6, Nitazoxanide (400 mg/kg).

On the 28^th^ day, blood samples were collected, and the animals were sacrificed using CO_2_. Following standard research methods, their hind paw limbs were sectioned and preserved in a 10% neutral formalin solution for further study.^[30]^

### Determination of Paw Volume

The measurement of paw volume was performed with a ple-thysmometer (Ugo Basile, Italy). The initial paw volume was documented on day 0, with follow-up measurements conducted on the 1^st^, 3^rd^, 7^th^, 11^th^, 14^th^, 21^st^, and 28^th^ days after the CFA injection.^[30]^

### Determination of Body Weight

The initial body weight was measured on day 0 before the CFA injection was given, following a one-week acclimatization period. Weight measurements were then taken 7^th^, 14^th^, 21^st^, and 28^th^ days after the CFA injection.^[30]^

### Total Arthritic Index

The total mean arthritic index was calculated by grading the secondary lesions using accepted scoring procedures (Supplementary Table 1). The tail, nose, ears, forepaws, and hind paws were examined at the end of the dosing period based on the following criteria.^[31]^

### Behavioural Parameters

*Stair Climbing Test*

A seven-day acclimatization phase was instituted before the experiment to evaluate the stair-climbing capabilities of the rats. The rats were kept in a wooden enclosure (30 cm high) with three varying elevation steps (5 cm, 10 cm, and 15 cm) during this phase. A water-filled petri dish was placed strategically on the second step, and food pellets were positioned on the third step to promote climbing behaviour. On the evaluation day, the rats experienced a 6-hour fasting interval, after which their climbing performance was evaluated using a standardized scoring system: Score 0, No climb of animal; Score 1, One stair climbing by the animal; Score 2, First and second stair climbing by the animal; Score 3, Three stairs climbing by the animal.^[32,33]^

*Motility Test*

The animals were acclimated for seven days before the administration of CFA. During this period, each rat was kept separately in a 100 cm by 50 cm by 50 cm wooden box with a climbing chamber. Motility assessments were conducted on the 1^st^, 3^rd^, 7^th^, 14^th^, 21^st^, and 28^th^ days. The motility of each rat was scored based on predefined criteria during these observations: Score 0, The rat is noted to be in a stationary posture; Score 1, By crawling, the rat demonstrates movement; Score 2: By walking, the rat demonstrates ambulation activities; Score 3: The rat showcases a climbing activity; Score 4: The rat engages in climbing and running repetitively; Score 5, The rat performs climbing and running with expertise.

These evaluations were performed following the established research methodology.^[32,33]^

### Assessment of Red Blood Cells (RBC), White Blood Cells (WBC), Platelets, and Haemoglobin (Hb)

On day 28, the rodents were euthanized, and blood samples were collected *via* retro-orbital puncture. These specimens were subsequently analyzed to determine platelet count, WBC count, RBC count, and Hb levels.^[32,33]^

### Assessment of STAT-3, NF-κB, TNF-α and IL-6 Levels

On day 28, the rats were euthanized, and blood samples were collected *via* retro-orbital puncture. The serum concentrations of TNF-α and IL-6 were measured. Additionally, the hind paws were excised, and the soft tissues were separated. Using a tissue homogenizer, a homogenate of phosphate-buffered saline (PBS) (pH 7.4) was prepared. The homogenate was centrifuged at 12, 000 rpm for 10 min, and the supernatant was analyzed for STAT-3 and NF-κB concentrations. These concentrations were quantified using kits obtained from Allianz Bio Innovation Pvt. Ltd., following the manufacturer’s protocol.^[34,35]^

### Histopathological Examination

Hind paw tissues from the rats were fixed in 10% buffered formalin, trimmed, and decalcified in 5% formic acid. The samples were then processed, embedded in paraffin, and sectioned into 5 μm slices for hematoxylin and eosin staining. The stained sections were examined under a light microscope at 100× magnification to observe any inflammatory changes resulting from normal control as well as in treatment groups.^[36]^

### Radiological Analysis

On the 28^th^ day, a radiographic analysis of the ankle joints was conducted. Anaesthetized rats were placed on radiography equipment, about 90 cm away from the X-ray source. The left hind paws were examined utilizing 40 kW exposures lasting 0.01 seconds. Radiographic scoring was based on articular cartilage injury, soft tissue swelling, and osteophyte growth. The following criteria were used to determine the scores: Score 0 indicated no harm; score 1 indicated mild damage; score 2 indicated considerable damage; and score 3 indicated serious damage.^[37]^

### Statistical Analysis

Data were expressed as mean ± standard error of the mean (SEM) and analysed using One-way ANOVA to determine statistical significance. Tukey’s post hoc test was conducted to identify the significant differences among groups, and Dunnett’s test was used for specific comparisons to the disease control group. Statistical analysis was performed using GraphPad Prism (9.5.1), with a *P*-value of < 0.05 was considered significant.

## Results

### Paw Volume

**Figure 1:**
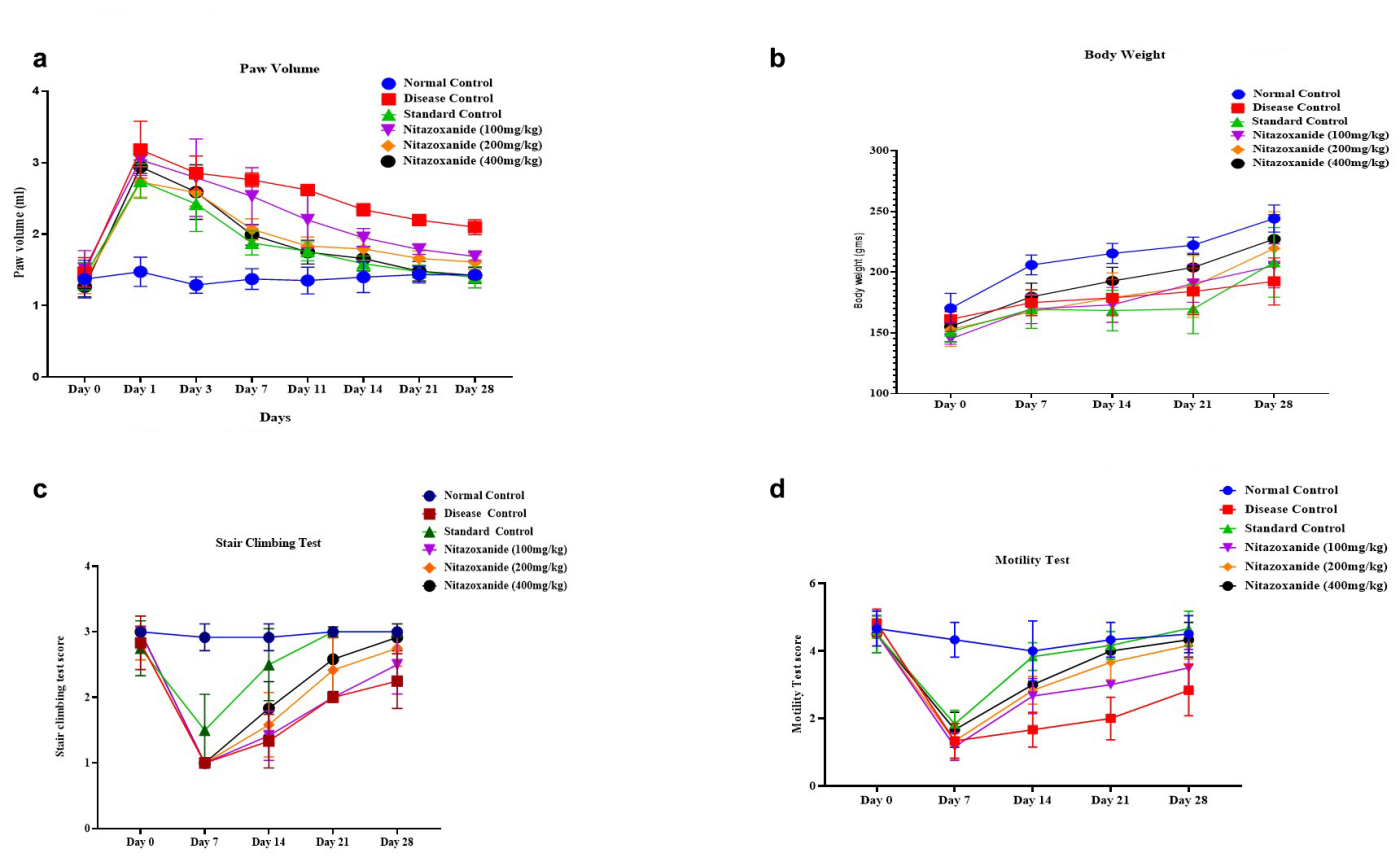
A, NTZ effect on left hind paw volume in CFA-induced arthritic rats. B, Average body weight in CFA-induced arthritic rats. C, Mean stair-climbing performance in CFA-induced arthritic rats and D Mean motility score in CFA-induced arthritic rats. The values are expressed as mean ± SEM. Data were analysed by one-way ANOVA followed by a post-hoc Tukey HSD test. (^###^P < 0.001 vs. Normal control, ***P < 0.001 vs. disease control, **P < 0.01 vs. disease control, and *P < 0.1 vs. disease control)

The measurement of paw volume in CFA-induced arthritic rats indicated significant results. A progressive increase in paw volume was observed, reflecting the advancement of arthritis over time. Prednisolone treatment demonstrated robust anti-inflammatory effects, leading to a notable reduction in paw volume. On day 14, nitazoxanide at a dose of 200 mg/kg exhibited measurable anti-inflammatory activity compared to the disease control group (*P* < 0.01). Furthermore, Nitazoxanide at 400 mg/kg showed significant anti-inflammatory effects across multiple time points, including days 7, 11, 14, 21, and 28 (*P* < 0.01; *P* < 0.001), with *F* = 8.186, as depicted in Figure 1a. The study’s results elucidated the progressive characteristics of CFA-induced arthritis and highlighted the clinical effectiveness of prednisolone and nitazoxanide in alleviating inflammation associated with rheumatoid arthritis.

### Body Weight

CFA-induced arthritis significantly affected body weight, with a pronounced decrease observed from days 7 to 28 compared to the control group (*P* < 0.01, *P* < 0.001), as shown in Figure 1b. In the initial phases, no notable alterations were observed; however, by the seventh day, variations in weight became apparent across all treatment cohorts. The administration of Prednisolone did not result in a statistically significant alteration in body weight when compared to the arthritic control group. Conversely, Nitazoxanide at dosages of 200 mg/kg and 400 mg/kg demonstrated substantial enhancements in body weight, with significant increases recorded on days 14 (*P* < 0.01), 21 (*P* < 0.01), and 28 (*P* < 0.001). By day 28, all treatment groups, including those receiving Prednisolone, exhibited significant and persistent increases in body weight (*P* < 0.001, *F* = 1.323). These findings highlight the severity of arthritis and suggest the promising potential of Nitazoxanide as a therapeutic agent for managing weight loss and inflammation associated with rheumatoid arthritis.

### Total Arthritic Index

The total mean arthritic index was evaluated by individually assessing arthritic scores for the nose, ears, tail, hind paws, and fore paws. CFA-induced disease group rats exhibited the highest total mean arthritic index at 5.16 ± 0.51. In contrast, treatment with NTZ and prednisolone significantly reduced the total mean arthritic index. Specifically, NTZ at 400 mg/kg resulted in a score of 3.66 ± 0.30 (*P* < 0.05), pred-nisolone achieved 2.83 ± 0.35, NTZ at 200 mg/kg recorded 3.91 ± 0.42 (*P* < 0.05), and NTZ at 100 mg/kg showed 4.5 ± 0.47. The percentage reductions in the total mean arthritic index compared to CFA-induced disease group rats were calculated as 30.0% for NTZ (400 mg/kg), 45.1% for pred-nisolone, 24.2% for NTZ (200 mg/kg), and 12.7% for NTZ (100 mg/kg). These results highlight the efficacy of NTZ, particularly at 400 mg/kg, in mitigating inflammation in arthritic rats (Supplementary Table 2).

### Behavioral Parameters

#### Stair Climbing Test

At the onset of the study (day 0), all treatment groups exhibited motility scores comparable to the normal control group, with no significant differences observed. However, as the investigation progressed, significant gains in motility were observed. By day 7, the group receiving prednisolone at a dose of 10 mg/kg had significantly improved motility compared to the CFA-induced arthritic disease control group (*P* < 0.05). On day 14, both the nitazoxanide treatment groups (400 mg/kg and 200 mg/kg) showed significant improvements in motility relative to the disease control (*P <* 0.01). By day 21, the group administered nitazoxanide at a dosage of 400 mg/kg demonstrated a significant enhancement in motility compared with the disease control group (*P* < 0.01, *F* = 7.955). These findings highlight the significant therapeutic efficacy of nitazoxanide, particularly at increased dosages, in mitigating the effects of CFA-induced arthritis on locomotion. The outcomes are illustrated in Figure 1c.

#### Motility Test

The analysis of motility among the different treatment groups demonstrated significant outcomes. At baseline (day 0), all groups exhibited comparable motility scores, with no significant differences from the normal control, indicating uniform baseline motility levels. By the 7th day, the group receiving prednisolone (10 mg/kg) revealed a pronounced improvement in motility relative to the disease control group (*P* < 0.05), highlighting the positive effects of prednisolone on mobility. On day 14, significant improvements in motility were observed in the NTZ 100 mg/kg and NTZ 200 mg/kg treatment groups compared to the disease control (*P* < 0.01). Notably, the NTZ 400 mg/kg group demonstrated a highly significant enhancement in motility (*P* < 0.001), emphasizing its therapeutic potential. By day 21, both NTZ 200 mg/kg and NTZ 400 mg/kg treatment groups showed significant motility improvements compared to the disease control (*P* < 0.001, *F* = 8.159). These results, depicted in Figure 1d, emphasize the evolving nature of motility alterations in reaction to arthritis and validate the effectiveness of prednisolone, NTZ 400 mg/kg, and NTZ 200 mg/kg treatments in enhancing mobility and reducing the effects of CFA-induced arthritis.

### STAT-3, NF-κB, TNF-α and IL-6 Levels

**Figure 2:**
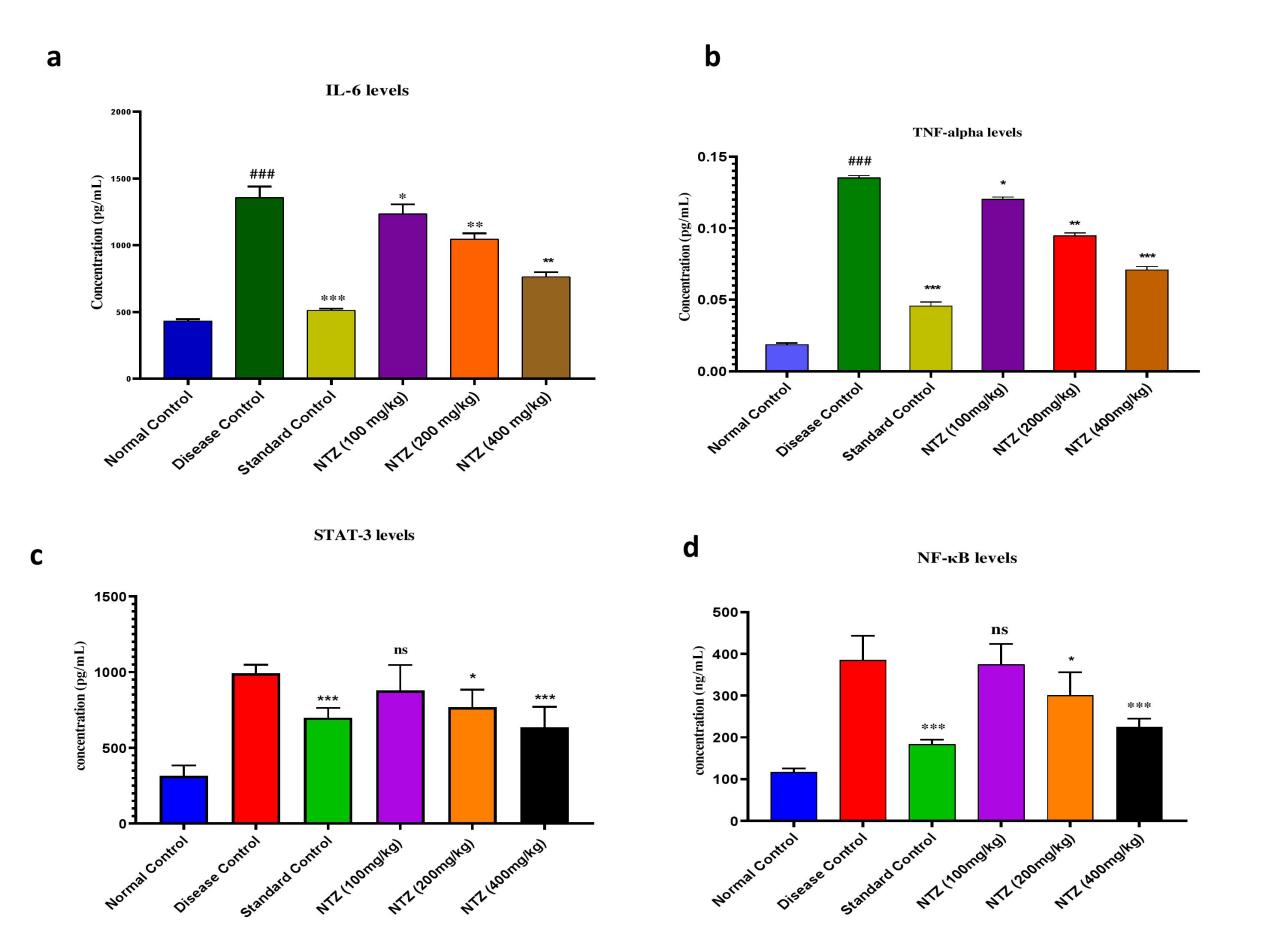
NTZ effect on pro-inflammatory cytokines in CFA-induced arthritic rats. (a) interleukin (IL-6) levels, (b) TNF-alpha levels, (c) STAT3 levels, and (d) NF-κB levels. The values are expressed as mean ± SEM. Data were analysed by one-way ANOVA followed by a post-hoc Tukey HSD test. (^###^P < 0.001 vs. Normal control, ***P < 0.001 vs. disease control, **P < 0.01 vs. disease control, and *P < 0.1 vs. disease control)

In our comprehensive study, we looked closely at how different therapies affected important inflammatory indicators in RA. Significant variations were noted in the concentrations of several important cytokines. Notably, NF-κB activity was inhibited only in the NTZ 400 mg/kg treatment group, indicating the potent anti-inflammatory effect of NTZ in this context (*P <* 0.001, *F* = 3.218). This suggests NTZ’s potential to modulate NF-κB, a crucial transcription factor in inflammation. In addition, the NTZ 400 mg/kg, NTZ 200 mg/kg, and prednisolone-treated groups had significantly lower levels of TNF-α, a key pro-inflammatory cytokine (*P* < 0.001, *F* = 0.6944). This reduction highlights NTZ’s ability to suppress TNF-α, a key mediator in arthritis inflammation. Similarly, significant reductions in IL-6 levels were observed in both the NTZ 400 mg/kg and prednis-olone-treated groups (*P* < 0.001, *F* = 5.236), reinforcing NTZ’s capacity to regulate this inflammatory cytokine. Additionally, NTZ at the 400 mg/kg dose significantly decreased STAT-3 levels (*P <* 0.001, *F* = 2.299), suggesting that NTZ may influence inflammatory signalling pathways involving STAT-3, further enhancing its anti-inflammatory profile. Collectively, these findings indicate that NTZ, particularly at the 400 mg/kg dose, effectively modulates several key inflammatory cyto-kines, including IL-6, TNF-α, NF-κB, and STAT-3, positioning it as a promising candidate for targeting immune responses in rheumatoid arthritis, as shown in Figure 2.

### RBC, WBC, Platelets, and Hb Levels

**Figure 3:**
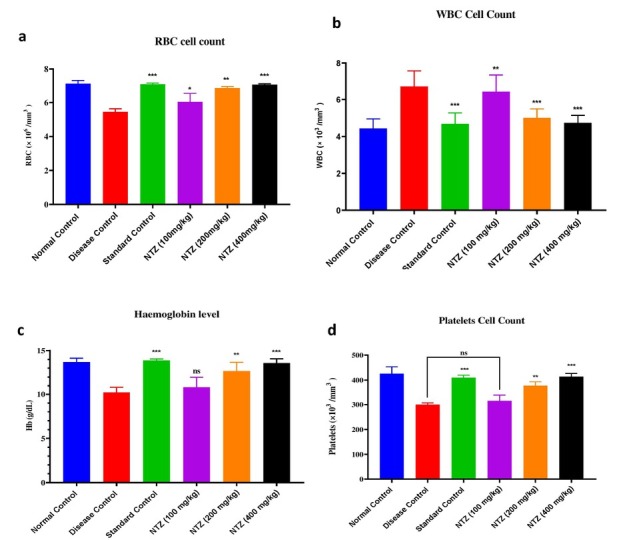
NTZ effect on haematological parameters across all groups, showing changes in (a) RBC levels, (b) WBC levels, (c) Hb levels, and (d) platelet levels. The values are expressed as mean ± SEM. Data were analyzed by one-way ANOVA followed by a post-hoc Tukey HSD test. ***P < 0.001 vs. disease control, **P < 0.01 vs. disease control, and *P<0.1 vs. disease control)

The effect of NTZ on haematological parameters was assessed using blood specimens collected on the 28th day. Significant changes were observed in comparison to the disease control group, particularly in the groups treated with NTZ and the Prednisolone 10 mg/kg. In the prednisolonetreated group, red blood cell (RBC) levels showed a notable increase, indicating enhanced erythropoiesis. Similarly, the NTZ 400 mg/kg treatment group exhibited a marked rise in RBC levels, reflecting improved hematopoietic activity. White blood cell (WBC) counts demonstrated a significant reduction in the NTZ 400 mg/kg group compared to the disease control. This decrease suggests that NTZ may have anti-inflammatory properties, as lower WBC counts often correlate with a reduction in systemic inflammation. Haemoglobin (Hb) levels also increased significantly in the NTZ 400 mg/kg group (*P* < 0.001, *F* = 2.144), indicating a positive effect on oxygen-carrying capacity and erythropoiesis. Platelet counts showed a substantial increase in the NTZ 400 mg/kg group compared to the disease control, suggesting that NTZ may influence platelet production or turnover. This increase in platelets, along with changes in other haematological parameters, points toward NTZ’s role in modulating both inflammatory responses and haematological functions. These findings suggest that NTZ, particularly at a dose of 400 mg/kg, and prednisolone significantly impacted haematological parameters, reflecting their potential to regulate inflammation and improve hematopoietic balance. The observed effects underscore NTZ’s therapeutic potential in inflammatory conditions such as rheumatoid arthritis (*P <* 0.001 *vs*. disease control, *P* < 0.01 *vs*. disease control, *P* < 0.1 *vs*. disease control; *F* = 1.146), as illustrated in Figure 3.

### Histopathology Examination

**Figure 4:**
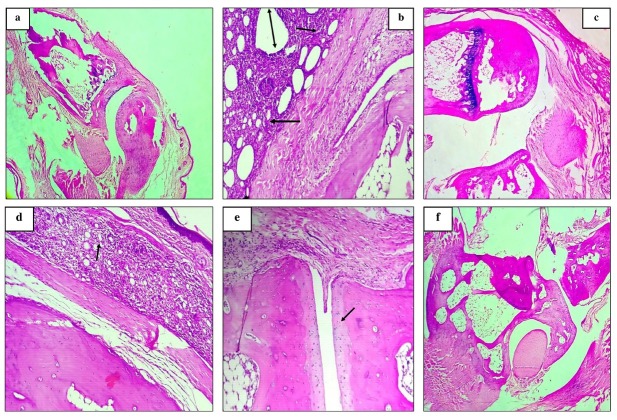
Histopathological examination of experimental groups (Images (a–f) represent (a) normal control, (b) disease group, (c) predniso-lone-treated group, (d) NTZ 100 mg/kg, (e) NTZ 200 mg/kg, and (f) NTZ 400 mg/kg.

Histopathological analysis using hematoxylin-eosin staining of ankle joints revealed distinct differences among treatment groups, allowing for the assessment of various therapeutic interventions on the progression of arthritis in experimental groups. Comparisons were made to both disease and normal control groups. The normal control group exhibited no abnormalities, maintaining a typical joint structure with smooth sy-novial cells arranged in a single layer. In contrast, the disease control group showed characteristic signs of arthritis, including pannus formation, mild synovial fibrosis, mild to moderate synovial inflammation, and minimal to mild cartilage erosion, resulting in a histopathological grading of 2 to 3. The disease control group (Figure 4b) demonstrated reduced joint space, significant infiltration of inflammatory cells, and pan-nus formation, which are key features of RA. In contrast, the therapeutic interventions led to notable improvements in joint structure. The prednisolone-treated group (c) exhibited partial recovery, with improved joint space, suggesting a reduction in synovial inflammation and cartilage erosion, though not complete restoration of normal joint morphology. Joints treated with NTZ at 100–200 mg/kg (d-e) displayed mild edema and inflammatory cell infiltration, with some residual inflammation and pannus, but also indicated a reduction in the severity of the condition. Remarkably, joints treated with NTZ at 400 mg/kg (f) showed near-normal joint morphology, characterized by restored joint space and smooth synovial lining. The severe pannus formation and synovial inflammation observed in the disease control group were significantly reduced in this high-dose NTZ group, highlighting the therapeutic efficacy of NTZ, especially at the 400 mg/kg dosage. The analysis demonstrated the progression of arthritis in the disease control group, where pannus formation and joint deterioration were prominent. Following treatment with NTZ and prednisolone, improvements in joint structure were observed. NTZ, particularly at the higher dosage, showed significant potential in reducing synovial inflammation and preserving joint integrity, suggesting promising therapeutic benefits for arthritis management (Supplementary Table 3).

### Radiological Analysis

**Figure 5:**
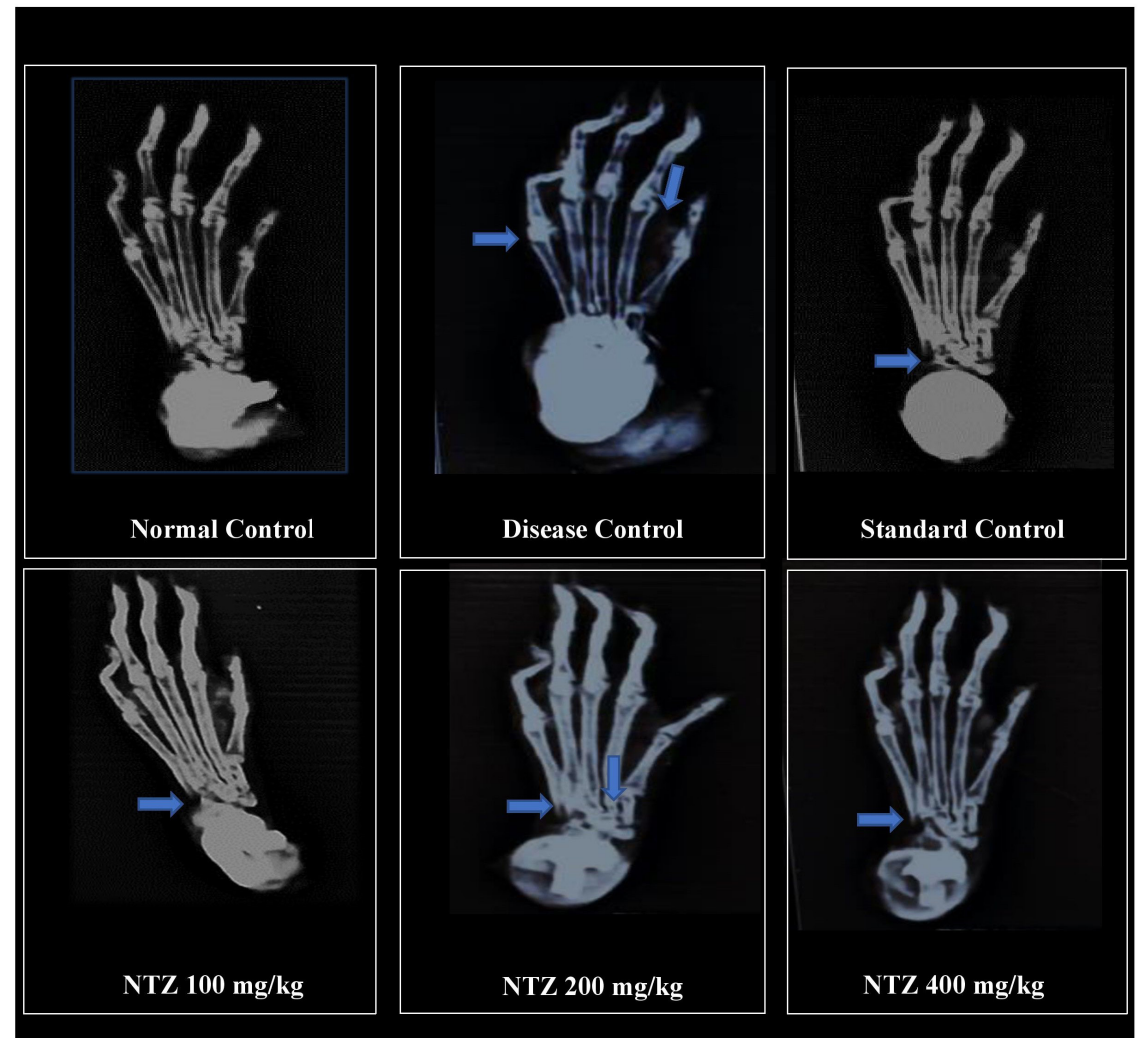
Radiological analysis of rat hind limbs across different groups. Blue arrows indicate regions affected by arthritis. NTZ: Nitazoxanide.

Radiological evaluation of hind limb joints in CFA-induced arthritic rats revealed significant pathological changes in the disease control group, including joint space narrowing, cartilage loss, bone erosion, soft-tissue oedema, and joint malformations, indicative of severe arthritis progression. In contrast, the Normal Control group displayed intact joint architecture without signs of damage. NTZ treatment showed dose-dependent improvements. The 100 mg/kg group exhibited partial preservation of joint structure and reduced inflammation, while the 200 mg/kg group demonstrated moderate protection against bone erosion and joint degeneration. The 400 mg/kg group showed substantial recovery, with near-complete preservation of joint integrity and minimal arthritis-related changes. The Prednisolone 10 mg/kg exhibited reduced radiological damage. These findings highlight NTZ’s therapeutic potential in alleviating joint degeneration, promoting cartilage repair, and protecting against arthritis-induced bone damage, as depicted in Figure 5, where arrows indicate areas of bone erosion and disturbances in joint integrity.

## Discussion

A variety of physiological mechanisms, including acute and chronic inflammation, as well as other immune responses, play a role in the development of RA. CFA-induced arthritis is characterized by rapid disease progression and is widely utilized for studying the underlying causes of RA and assessing potential treatments. Adjuvant arthritis is initiated by bacterial components in CFA, such as peptidoglycans and muramyl dipeptides.^[38,39]^ The current clinical approach to managing RA heavily relies on aggressive treatment strategies and potent pharmacological regimens, which come with significant side effects. Over the past decade, immunotherapy has also emerged as a potential option for RA treatment, although its clinical benefits are not yet fully understood. As a result, the exploration and development of alternative treatments for RA have gained considerable attention, demonstrating great success due to their multi-faceted therapeutic effects.^[40]^ However, about 5%–20% of RA patients remain resistant to all available treatments, and 40% do not respond to specific drugs.^[41]^

There is an urgent need to discover new agents to control disease progression in RA patients. The STAT-3, a key downstream signalling component, has been recognized for 30 years and plays a role in mediating various physiological and pathological responses.^[42]^ A previous study demonstrated that NTZ inhibited receptor activator of nuclear factor kappa-B ligand (RANKL)-induced osteoclastogenesis in primary bone marrow-derived macrophage cells in mice and prevented bone loss caused by ovariectomy *in vivo* by disrupting STAT-3 activation and nuclear factor of activated T cells c1 (NFATc1) expression.^[26]^ However, the potential role of NTZ in other bone-destructive diseases remains uncertain. Current research suggests that TNF-α and IL-6 play a role in cartilage destruction and synovial proliferation, with elevated IL-6 levels contributing to joint damage due to its abundance in the rheumatoid synovium. In this study, similar results were observed in CFA-induced rats, where treatment with NTZ effectively reduced the levels of inflammatory cytokines.

The CFA-induced RA model is widely used and highly effective because it closely mimics the characteristics of human RA, making it valuable for studying anti-arthritic agents.^[43,44]^ Joint edema is commonly observed following inflammation and is considered a characteristic hallmark of RA.^[45]^ The results indicated that NTZ demonstrated a protective effect against CFA-induced RA, as shown by reduced paw volume, improved body weight, and lower arthritic scores. Additionally, NTZ alleviated inflammatory conditions by suppressing oxidative stress and inflammation. During RA, there is a sustained decline in body weight attributed to the compromised absorption of leucine and glucose within the gastrointestinal tract.^[8,10]^ In the current investigation, a notable decrease in body weight was documented in RA rats, whereas NTZ administration substantially enhanced body weight, suggesting a beneficial influence on the absorption of glucose and leucine within the intestinal tract. Alterations in haematological parameters are commonly observed during RA.^[5]^ During RA, a reduction in RBC count is observed due to the development of an anaemic condition. This situation arises from the deformability of erythrocytes.^[46, 47, 48]^ Additionally, Hb levels also decrease due to reduced erythropoietin production in the bone marrow and the destruction of immature RBC.^[6]^ In the study, CFA-induced rats exhibited significant alterations in haematological parameters. However, NTZ-treated rats showed modulation of these parameters, suggesting its potential in mitigating RA-related haematological disturbances.

Cytokines play a critical role in mediating the inflammatory response in knee and ankle joints. They contribute to bone and articular cartilage damage, which leads to inflammation in the synovial tissue and triggers other pathological changes associated with joint damage.^[49,50]^ IL-6 plays a crucial role in the expansion and progression of RA in humans and is involved with various inflammatory cells. Levels of TNF-α and NF-κB significantly increase, which activates inflammatory cells and drives the inflammatory response. These alterations initiate multiple signalling pathways, contributing to the persistence of inflammation, tissue damage, and the progression of the disease.^[51,52]^ CFA-induced rats exhibited an increase in cytokine levels, while NTZ treatment significantly (*P* < 0.001) suppressed the levels of TNF-α, NF-κB, STAT-3, and IL-6.

The ankle joint’s histopathological and radiographic evaluations emphasize the metatarsals and phalanges within the NTZ (400 mg/kg) group, revealing a reduction in arthritis. These findings suggested that NTZ inhibits the STAT-3 and NF-κB pathways, modulating cellular function and contributing to the attenuation of arthritis. Additionally, other inflammatory mediators and pathways, such as Interferon-gamma (IFN-γ), Cyclooxygenase-2 (COX-2), and IL-10, could be further explored to understand the broader anti-inflammatory mechanisms of NTZ.

A recent study by Li *et al*. evaluated the therapeutic effects of NTZ in a CIA (Collagen-Induced Arthritis) mouse arthritis model. Their findings showed that NTZ inhibited the proliferation, migration, and invasion of Rheumatoid arthritis Fibroblast-Like Synoviocytes (RA-FLS) in a dose-dependent manner, without inducing apoptosis. NTZ reduced the mRNA expression of IL-1β, IL-6, Receptor Activator of Nuclear Factor *kappa* B Ligand (RANKL), and TNF-α-induced transcription of these cytokines, through the inhibition of the Janus kinase 2 (JAK2)/STAT-3 and NF-κB pathways, without affecting mi-togen-activated protein kinase (MAPK) or STAT-3 phosphory-lation. Additionally, NTZ reduced synovial inflammation and bone erosion in CIA mice by decreasing inflammatory mediator production and osteoclast formation, supporting its potential as an anti-inflammatory and anti-erosive agent for RA.^[53]^ In contrast, the present study used a CFA-induced arthritis model in rats and found that NTZ similarly lowered serum TNF-α and IL-6 levels, reducing synovitis, pannus formation, and bone/cartilage destruction in histological analyses of the toe joints. However, CFA induces a systemic inflammatory response with enhanced adaptive immune activity and elicits immunity against mycobacterial antigens, it differs from the CIA model, which primarily stimulates the innate immune system. Despite these differences, both studies demonstrated NTZ’s ability to modulate inflammation effectively.^[54]^ Li *et al*. also reported that NTZ inhibited STAT-3-T705 phosphoryla-tion, an inhibitor of nuclear factor *kappa* B (IκBα), and p65, indicating suppression of the STAT-3 and NF-κB pathways. In the present study, NTZ inhibition of IL-6 was linked to STAT-3 and NF-κB deactivation in CFA-induced paw tissues, reducing inflammation. Comparing these findings on STAT-3 and NF-κB modulation across models will provide insights into NTZ effects on these pathways and clarify the molecular mechanisms behind its therapeutic action. Such comparative studies could significantly enhance our understanding of NTZ role in inflammation and further elucidate its therapeutic potential in RA. By exploring how NTZ modulates key pathways like STAT-3 and NF-κB across different models, researchers can gain valuable insights into its mechanism of action, potentially leading to more targeted and effective treatments for RA.

The limitations noted in the research accentuate the critical need for further extensive preclinical and clinical assessments to confirm the therapeutic efficacy of NTZ and establish the ideal dosing for human use. Based on the findings, the studies suggested that NTZ effects are dose-dependent, which can inform dosing strategies in clinical trials. The modulation of key inflammatory pathways, such as STAT-3 and NF-κB, indicates that different doses of NTZ may impact these pathways differently, affecting inflammation and clinical outcomes. Given that NTZ demonstrated anti-inflammatory and anti-erosive effects in animal models, clinical trials can use these insights to determine the optimal dose range, balancing efficacy with safety. This would help establish the most effective and safe dosing regimen for treating RA in humans. When comparing NTZ to Prednisolone; RA treatments like methotrexate (MTX) and JAK inhibitors, there are notable differences in safety profiles. JAK inhibitors are associated with an increased risk of infectious adverse events (especially viral, fungal, and mycobacterial), musculoskel-etal and connective tissue disorders, embolism, thrombosis, and neoplasms, with tofacitinib linked to a higher incidence of gastrointestinal perforations.^[55]^ In contrast, MTX may be limited by side effects such as interstitial lung disease (ILD), hepatotoxicity, myelosuppression, and infections, requiring constant vigilance.^[56]^ On the other hand, NTZ has been used safely by over 500 million people worldwide since its introduction 25 years ago, demonstrating a favourable safety profile in both adults and children.^[57]^ This comparison highlights NTZ’s potential as a promising therapeutic option with a better safety record than current treatments. Additionally, the study lacks a comprehensive evaluation of RA-specific biomarkers, which could provide more detailed information on its impact on disease activity and progression. The relatively short duration of the experiment may also be inadequate for observing long-term outcomes or potential side effects. Overcoming these challenges in upcoming research projects would facilitate the development of a more solid framework for determining the impact of NTZ as a treatment for RA.

## Conclusions

Our findings strongly suggested that Nitazoxanide exhibits significant anti-arthritic activity in CFA-induced arthritis by regulating key pro-inflammatory cytokines. This modulation leads to a reduction in intracellular levels of STAT-3 and NF-κB, a critical mediator in the inflammatory response. Given its ability to influence these pathways, Nitazoxanide shows promise as an effective alternative or adjunct treatment for arthritis. However, further studies are necessary to explore its potential in clinical settings and to gain a comprehensive understanding of its therapeutic mechanism.

## Supplementary Material

Supplementary Material Details
