# Supplementary material for: Nitazoxanide alleviates CFA-induced rheumatoid arthritis in Wistar rats by modulating the STAT-3 and NF-κB pathways
Source: Rheumatol Immunol Res. 2025 Apr 2;6(1):29–41. doi: 10.1515/rir-2025-0004 (PMC11966199; doi:10.1515/rir-2025-0004)
Supplement: Supplementary file 1 — Supplementary Material Details [file rir-2025-0004_sm.pdf]

Supplementary Table 1. Scoring system for arthritic index

Lesion site	Nature of lesion	Score
Ears	Absence of nodules<br>Presence of nodules	0<br>1
Nose	Absence of swelling of connective tissue<br>Presence of swelling of connective tissue	0<br>1
Tail	Absence of nodules<br>Presence of nodules	0<br>1
Fore paws	Absence of inflammation<br>Presence of inflammation at least in one joint	0<br>1
Hind paws	Absence of inflammation<br>Slight inflammation<br>Moderate inflammation<br>Marked/severe inflammation	0<br>1<br>2<br>3

Supplementary Table 2. Effect of Different Treatments on the Total Mean Arthritic Index

Groups	Total mean arthritic index	Percentage change in arthritic index as compared to disease control
Disease Control	5.16 ± 0.51	-
Standard Control	2.83 ± 0.35**	45.1
NTZ (100 mg/kg)	4.5 ± 0.47	12.7
NTZ (200 mg/kg)	3.91 ± 0.42*	24.2
NTZ (400 mg/kg)	3.66 ± 0.30**	30.0

All values are expressed as mean ± SEM, $n = 6$, one-way ANOVA followed by Dunnett Post hoc and multiple comparison test. *$P < 0.01$ as compared to disease control, **$P < 0.001$ as compared to disease control. NTZ: Nitazoxanide.

Supplementary Table 3. Scoring for examination of rats of hind limb of different groups

Groups	Hyperaemia / congestion	Edema	Inflammatory cell infiltration	Synovial changes	Cartilage changes	Bone changes	Grade of inflammation
Normal Control	Nil	Nil	Nil	Not seen	Not seen	Not seen	Nil
Disease Control	+	+	+ to ++	Not seen	Not seen	Not seen	2 to 3
Standard Control	Nil	Nil	Nil	Not seen	Not seen	Not seen	Nil
NTZ (100 mg/kg)	Nil	++	+	Not seen	Not seen	Not seen	1 to 2
NTZ (200 mg/kg)	Nil	+	+	Not seen	Not seen	Not seen	0 to 1
NTZ (400 mg/kg)	Nil	Nil	Nil	Not seen	Not seen	Not seen	Nil

NTZ: Nitazoxanide.
